# Foot-and-mouth disease virus leader proteinase: Structural insights into the mechanism of intermolecular cleavage

**DOI:** 10.1016/j.virol.2014.08.023

**Published:** 2014-11

**Authors:** Jutta Steinberger, Irina Grishkovskaya, Regina Cencic, Luiz Juliano, Maria A. Juliano, Tim Skern

**Affiliations:** a Max F. Perutz Laboratories, Medical University of Vienna, Dr. Bohr-Gasse 9/3, A-1030 Vienna, Austria; b Max F. Perutz Laboratories, University of Vienna, Department of Structural and Computational Biology, Campus Vienna Biocenter 5, A-1030 Vienna, Austria; c Department of Biophysics, Escola Paulista de Medicina, Universidade Federal de São Paulo, Rua Três de Maio 100, 04044-20 São Paulo, Brazil

**Keywords:** FMDV, foot-and-mouth disease virus, Lb^pro^, leader proteinase, sLb^pro^, shortened leader proteinase (lacking 6 C-terminal amino acids), wt, wildtype, CTE, C-terminal extension, eIF, eukaryotic initiation factor, Papain-like cysteine proteinase, Host cell shut-off, Active site, Polyprotein processing, Substrate binding, Initiation of protein synthesis

## Abstract

Translation of foot-and-mouth disease virus RNA initiates at one of two start codons leading to the synthesis of two forms of leader proteinase L^pro^ (Lab^pro^ and Lb^pro^). These forms free themselves from the viral polyprotein by intra- and intermolecular self-processing and subsequently cleave the cellular eukaryotic initiation factor (eIF) 4G. During infection, Lb^pro^ removes six residues from its own C-terminus, generating sLb^pro^. We present the structure of sLb^pro^ bound to the inhibitor E64-R-P-NH_2_, illustrating how sLb^pro^ can cleave between Lys/Gly and Gly/Arg pairs. In intermolecular cleavage on polyprotein substrates, Lb^pro^ was unaffected by P1 or P1′ substitutions and processed a substrate containing nine eIF4GI cleavage site residues whereas sLb^pro^ failed to cleave the eIF4GI containing substrate and cleaved appreciably more slowly on mutated substrates. Introduction of 70 eIF4GI residues bearing the Lb^pro^ binding site restored cleavage. These data imply that Lb^pro^ and sLb^pro^ may have different functions in infected cells.

## Highlights

• The leader proteinase (L^pro^) of foot-and-mouth disease virus is a virulence factor.
• L^pro^ can exist in three different forms in the infected cell.
• Structural analysis reveals how L^pro^ can accept basic residues at P1 and P1′.
• Isoform lacking six C-terminal residues is impaired in intermolecular cleavage.
• Properties of the isoforms may modulate enzymatic activity during viral replication.

## Introduction

**Fig. 1:**
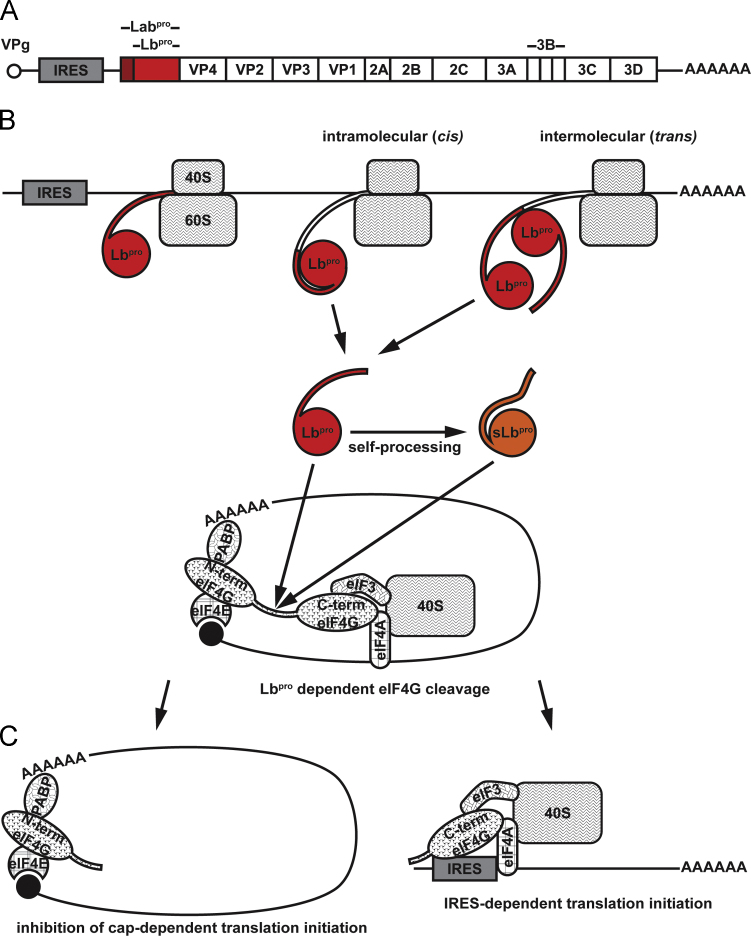
Schematic drawing of FMDV L^pro^ self-processing and eIF4G cleavage. (A) The FMDV RNA genome is shown as a black line, the single open reading frame as a box with the names of the mature proteins and the position of the IRES. L^pro^, being expressed either as Lab^pro^ or Lb^pro^, is indicated in red. (B) Synthesis of the polyprotein from the FMDV genome showing that Lb^pro^ can either be freed by an intramolecular or intermolecular reaction. sLb^pro^ (shown in orange) is generated by self-processing at the C-terminus of Lb^pro^. (C) The effect of eIF4G cleavage by Lb^pro^ or sLb^pro^. The cellular mRNA is shown as a black line with the cap structure as a filled circle. Lb^pro^ and sLb^pro^ are shown in red and orange, respectively. The 40S ribosomal subunit, the polyA-binding protein (PABP), eIF4G, eIF4E, eIF4A and eIF3 are shown in different shades of grey. Following cleavage of eIF4G by Lb^pro^ or sLb^pro^, the capped mRNA is no longer connected to the 40S subunit and cannot be translated. In contrast, the viral RNA can bind to the C-terminal fragment of eIF4G and thus to the 40S subunit via eIF3.

Virally encoded proteinases play essential roles not only in the processing of the viral proteins but also in cleavage of host cell proteins in order to manipulate cellular processes to the advantage of the virus. One of the first such reactions to be documented was the modification of cellular translation factors during picornaviral replication leading to the shut-off of protein synthesis from capped cellular mRNA (Etchison et al., 1982, Leibowitz and Penman, 1971). This reaction was subsequently shown to be performed by the 2A proteinase (2A^pro^) in enteroviruses (Kräusslich et al., 1987), a chymotrypsin-like cysteine proteinase (Petersen et al., 1999), whereas in aphthoviruses, the proteolysis is performed by the leader proteinase (L^pro^, illustrated in Fig. 1) (Devaney et al., 1988), a papain-like cysteine proteinase (Guarn**é** et al., 1998). The targets of both proteinases are the two homologues of the host protein eukaryotic initiation factor (eIF) 4G (Gingras et al., 1999). Cleavage of the eIF4G homologues prevents recruitment of capped mRNAs to the ribosome (Lamphear et al., 1995) whereas viral RNA can still be translated under these conditions as it initiates via an internal ribosome entry segment (IRES) (Martinez-Salas and Ryan, 2010). In addition, L^pro^ has been shown to be involved in impairing the host innate immune defence by influencing NF-κB activation and to have deubiquitinase activity (de Los Santos et al., 2007, de los Santos et al., 2009, Skern and Steinberger, 2014).

**Fig. 2:**
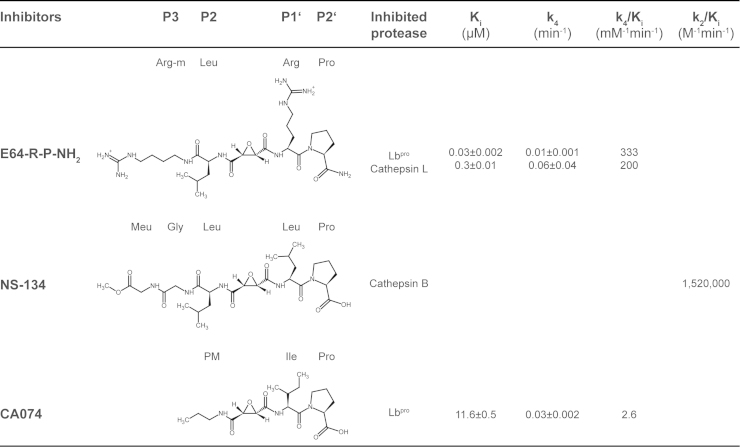
Chemical structures of inhibitors referred to in this work. The structures and kinetic parameters of the inhibitor E64-R-P-NH_2_ (Nogueira Santos et al., 2012) crystallised with sLb^pro^ are shown together with those of the inhibitors NS-134 (Stern et al., 2004) and CA074 (Yamamoto et al., 1997) whose structures were determined in complex with cathepsin B. The correspondence of side-chains in the inhibitors to substrate side-chains is shown using the nomenclature of Schechter and Berger, (19670.

Given these involvements in such different reactions as intramolecular and intermolecular self-processing, eIF4G cleavage and deubiquitination, it is not surprising that L^pro^ has unusual specificity determinants. These are well illustrated by the sequences of the three L^pro^ cleavage sites that have been determined directly by protein sequencing: KVQRKLK⁎GAGQSS for both intra- and intermolecular cleavage on the viral polyprotein between the C-terminus of L^pro^ and VP4 (Strebel and Beck, 1986), PSFANLG⁎RTTLST on eIF4GI (Kirchweger et al., 1994) and VPLLNVG⁎SRRSQP on eIF4GII (Gradi et al., 2004). Studies on L^pro^ intramolecular self-processing and cleavage of peptide substrates have revealed that L^pro^ can cleave before or after basic residues provided that the other amino acid before or after the scissile bond is glycine (Glaser et al., 2001, Nogueira Santos et al., 2012, Santos et al., 2009). However, a peptide that contained basic residues before and after the scissile bond was refractory to cleavage and was subsequently shown to be an inhibitor in the micromolar range (Santos et al., 2009). This information was then used to develop a nanomolar epoxide inhibitor based on E64, termed E64-R-P-NH_2_ (Santos et al., 2009); the structure and inhibitor parameters are shown in Fig. 2, together with those of the other inhibitors used or referred to in this work. The slow formation of the tight enzyme–inhibitor complex indicates that inhibition follows slow-binding kinetics (Santos et al., 2009, Zhou et al., 1998).

The structural basis for this unusual specificity has not been elucidated, as the present structures determined by X-ray crystallography and NMR (Cencic et al., 2007, Guarné et al., 1998, Guarné et al., 2000, Steinberger et al., 2013) only provide information on the S binding region but not on the S′ binding region of L^pro^. The nomenclature for sites (S) on the enzyme binding to residues of substrate (P) is that of Schechter and Berger (1967); prime site residues are those C-terminal to the scissile bond. Indeed, information on the nature of the S′ region from related papain-like proteinases is also sparse (Turk et al., 2012), with structural information only being available for cathepsin B (Stern et al., 2004, Turk et al., 1995, Yamamoto et al., 1997) determined with inhibitors similar to E64-R-P-NH_2_ (Fig. 2). However, cathepsin B is also unusual in being an exopeptidase, with an occluding loop that prevents access beyond the S2′ site, that is the site on the enzyme interacting with the P2′ residue of the substrate (Stern et al., 2004). Thus, any information on the S′ binding region of FMDV L^pro^ will shed light on the nature of this region in papain-like cysteine proteinases generally.

Understanding of the mechanism of L^pro^ is complicated by the presence of different forms of the protein in the infected cell (Sangar et al., 1987, Sangar et al., 1988). Two isoforms, Lab^pro^ and Lb^pro^ (Fig. 1), arise from the presence of two in-frame AUG codons for the initiation of protein synthesis on the viral RNA (Sangar et al., 1987). Consequently, the Lab^pro^ possesses an additional 28 amino acids at the N-terminus than Lb^pro^. Cao et al. (1995) demonstrated in cell culture that Lb^pro^ was essential whereas Lab^pro^ was not; nevertheless, there may still be as yet unknown roles for Lab^pro^ during infection in the host organism. In addition, a shortened form of Lb^pro^ (sLb^pro^) lacking 6 or 7 amino acids at the C-terminus has long been known (Sangar et al., 1988). The truncation arises through Lb^pro^ self-cleavage (Sangar et al., 1988) and can be observed in vitro when Lb^pro^ expressed in rabbit reticulocyte lysates (RRLs) is incubated for longer time periods (e.g. 1 h)(Mayer et al., 2008). A separate function for sLb^pro^ has not been identified; however, one report suggested that Lb^pro^ and sLb^pro^ may differ in their cleavage efficiencies in intermolecular cleavage of the polyprotein substrate (Cencic et al., 2007).

One clear difference between Lb^pro^ and sLb^pro^ is the ability of Lb^pro^ to form homodimers through interactions of the C-terminal extension (CTE) of one monomer and the substrate binding site of the neighbouring one and vice versa. sLb^pro^ cannot form homodimers in this way because it lacks the six most C-terminal residues. The Lb^pro^ homodimer has been observed by X-ray crystallography and NMR (Cencic et al., 2007, Guarné et al., 1998, Guarné et al., 2000, Steinberger et al., 2013) with the K_D_ being estimated from NMR analyses to be in the millimolar range (Cencic et al., 2007). Therefore, formation of the homodimer at concentrations of Lb^pro^ achieved when it is synthesised in the infected cell seems unlikely unless there is a high local concentration. In contrast, both Lb^pro^ and sLb^pro^ use an exosite featuring residues Tyr183 to Leu188 as well as Cys 133 to recognise binding sites located on the eIF4G homologues, located in both cases 20 to 30 amino acids from the cleavage site (Foeger et al., 2005). How this binding favours Lb^pro^ or sLb^pro^ cleavage of the eIF4G homologues is not known.

To investigate further the properties of sLb^pro^, we set out to determine the structure of sLb^pro^ complexed with the inhibitor E64-R-P-NH_2_ and to define differences in the cleavage of intermolecular polyprotein substrates by sLb^pro^ and Lb^pro^.

## Materials and methods

### Materials

The bacterial expression plasmid pET-11d sLb^pro^ (FMDV residues 29–195) was created by site-directed PCR mutagenesis of pET-11d sLb^pro^ C51A, described earlier (Guarné et al., 1998, Kirchweger et al., 1994), to restore the catalytic cysteine.

The plasmids that were used as templates for in vitro transcription pCITE-1d Lb^pro^ (residues 29–201 of Lb^pro^), pCITE-1d sLb^pro^ (residues 29–195 of Lb^pro^) and pCITE-1d Lb^pro^ C51A VP4/VP2 (residues 29–201 of Lb^pro^, all 85 residues of VP4 and 78 residues of VP2) have been described (Glaser et al., 2001). The constructs pCITE-1d Lb^pro^ C51A VP4/VP2 containing the mutations at position P1 and P1′ of the Lb^pro^-VP4 cleavage site (VQRKLG⁎RAGQ, VQRKLK⁎RAGQ, VQRKLG⁎AAGQ) were created by site-directed PCR mutagenesis of pCITE-1d Lb^pro^ C51A VP4/VP2. The construct pCITE-1d Lb^pro^ C51A VP4/VP2 containing the eIF4GI sequence SFANLG⁎RTTL at the Lb^pro^-VP4 cleavage site, termed pCITE-1d Lb^pro^ C51A VP4/VP2 SFANLG⁎RTTL (FMDV residues 29–195 of Lb^pro^, residues 669–678 of eIF4GI, residue 5–85 of VP4 and 78 residues of VP2), has been described (Cencic et al., 2007). The construct pCITE-1d Lb^pro^ C51A VP4/VP2 containing residues 599–678 of eIF4GI, termed pCITE-1d Lb^pro^ C51A eIF4GI_599-668_ VP4/VP2 SFANLG⁎RTTL, (FMDV residues 29–195 of Lb^pro^, residues 599–678 of eIF4GI, residue 5–85 of VP4 and 78 residues of VP2) was created by PCR amplification of residues 599–678 of eIF4GI using the plasmid pKS eIF4GI 400–739 as template and cloning of this fragment into pCITE-1d Lb^pro^ C51A VP4/VP2 via the restriction sites *Bpu10*I and *Sac*I.

The inhibitor E64-R-P-NH_2_ was prepared as described (Nogueira Santos et al., 2012).

### Protein expression and purification

Protein expression and purification were performed as described by Steinberger et al. (2013) with the following modifications. Proteins were expressed from the construct pET-11d sLb^pro^ transformed into BL21(DE3)LysE bacteria. To avoid degradation of the active protease, all purification steps were carried at a maximum of 10 °C.

### Preparation of the sLb^pro^-E64-R-P-NH_2_ complex

Purified sLb^pro^ was incubated with a fivefold molar excess of E64-R-P-NH_2_ over night at 4 °C to allow complex formation. Subsequently, the complex was dialysed against a buffer containing 50 mM NaCl, 10 mM Tris HCl pH 8, 1 mM TCEP, 5% glycerol to remove excess inhibitor. The concentration was adjusted to 18 mg/ml and centrifuged at 18,000*g* for 10 min at 4 °C to remove precipitated protein.

### Crystallisation, data collection, structure determination and refinement

Crystals of the sLb^pro^-E64-R-P-NH_2_ complex were initially obtained in the Wizard I and II screen crystallisation screen (Emerald Bio), using the sitting-drop vapour diffusion technique and a nanodrop-dispensing robot (Phoenix RE; Rigaku Europe, Kent, United Kingdom), and optimised to 0.1 M sodium acetate pH 4.8, 0.9 M NaH_2_PO_4_ and 1.2 M K_2_HPO_4_ using the hanging drop vapour diffusion technique at 22 °C and seeding technique. The seed stock was produced by a “seed-bead” kit from Hampton Research (Luft and DeTitta, 1999). The crystals were flash-frozen in liquid nitrogen in a reservoir solution supplemented with 25% glycerol prior to data collection.

Diffraction data sets were collected at the ESRF Synchrotron (Grenoble) at beamline ID14-1 at 100 K using a wavelength of 0.93 Å to 1.6 Å resolution, processed using the XDS package (Kabsch**,** 2010), converted to mtz format using POINTLESS and scaled with SCALA (Winn et al., 2011).

The crystal structure was solved by difference Fourier techniques using the protein atomic coordinates of the inactive mutant of sLb^pro^ from the Protein Data Bank (accession code 1QMY). Model building and refinement steps were performed with REFMAC and COOT. The structure was refined using the programs REFMAC (Murshudov et al., 1997) and Phenix Refine (Adams et al., 2010) and model building was done with the program Coot (Emsley and Cowtan, 2004). Data collection and refinement statistics are shown in Table 1. Stereo-chemistry and structure quality were checked using the MolProbity web server (Davis et al., 2007).

**Table 1:** X-ray parameters and refinement statistics.

**Data collection**	
Source	ID14-1, ESRF
Wavelength (Å)	0.93
Resolution (Å)	45.35–1.6 (1.69–1.6)^a^
Space group	P2_1_
Unit cell (Å, °)	*a*=45.81 *b*=110.68, *c*=56.77
	*α*=*γ*=90, *β*=98.12
Molecules / a.u.	3
Unique reflections	72906 (10232)
Completeness (%)	99.0 (95.0)
*R*_merge_^b^	0.037 (0.174)
*R*_meas_^c^	0.041 (0.213)
Multiplicity	4.9 (2.9)
I/sig(I)	29.9 (5.6)
B_Wilson_ (Å^2^)	22.5
**Refinement**	
*R*_cryst_^d^/ R_free_^e^	16.9/20.1
R.m.s.d. bonds (Å)	0.011
R.m.s.d. angles (°)	1.4
Ramachandran plot (%)	
favored/allowed/outliers	96.9/3.1/0

a Values in parentheses are for the highest resolution shell.

b $R_{merge}=\frac{\sum_{hkl}\sum_{i=1}^{N}\left|I_{i(hkl)}-{\bar{I}}_{\left(hkl\right)}\right|}{\sum_{hkl}\sum_{i=1}^{N}I_{i(hkl)}}$

c $R_{meas}=\frac{\sum_{hkl}\sqrt{N/\left(N-1\right)}\sum_{i=1}^{N}\left|I_{i(hkl)}-{\overset{¯}{I}}_{\left(hkl\right)}\right|}{\sum_{hkl}\sum_{i=1}^{N}I_{i(hkl)}}$ where ${\bar{I}}_{\left(hkl\right)}$ is the mean intensity of multiple $I_{i(hkl)}$ observations of the symmetry-related reflections, *N* is the redundancy

d $R_{cryst}=\frac{\sum \left|\left|F_{obs}\right|-\left|F_{calc}\right|\right|}{\sum \left|F_{obs}\right|}$

e *R*_free_ is the cross-validation *R*_factor_ computed for the test set of reflections (5%) which are omitted in the refinement process.

### In vitro transcription and translation

In vitro transcription reactions were performed as described (Neubauer et al., 2013) with the following modifications. The plasmids were cleaved with *BamH*I for the expression of active proteinases (pCITE-1d Lb^pro^ and pCITE-1d sLb^pro^) and *Sal*I for the substrates (pCITE-1d Lb^pro^ C51A VP4/VP2 and derivatives thereof).

In vitro translation reactions were performed as described (Cencic et al., 2007) with the following modification. To translate substrate proteins and proteinases, RNA was added to the reaction at concentrations of 14 ng/µl.

### Electrophoresis and immunoblotting

Electrophoresis and immunoblotting for protein analysis were performed as described (Neubauer et al., 2013), except for the separation of translation products when SDS-PAGE gels containing 17.5% acrylamide were used (Dasso and Jackson, 1989).

### Structural comparisons

Structural alignments and superimpositions were done using Coot (Emsley and Cowtan, 2004, Krissinel and Henrick, 2004). All drawings were created using PyMOL (DeLano**,** 2002). The electrostatic potential of sLb^pro^ was calculated using the Adaptive Poisson-Boltzmann Solver package (Baker et al., 2001) within PyMOL.

### Accession numbers

Coordinates for the structure determined here have been deposited in the protein data bank (pdb accession code 4QBB). The PDB identifiers of the structures used for comparisons were 1QOL for Lb^pro^, 1GEC for glycyl endopeptidase-complex with benzyloxycarbonyl-leucine-valine-glycine-methylene, 3CH3 for SERA5 from plasmodium falciparum, 1SP4 for bovine cathepsin B-complex with NS-134, 1QDQ for bovine cathepsin B-complex with CA074.

## Results and discussion

**Fig. 3:**
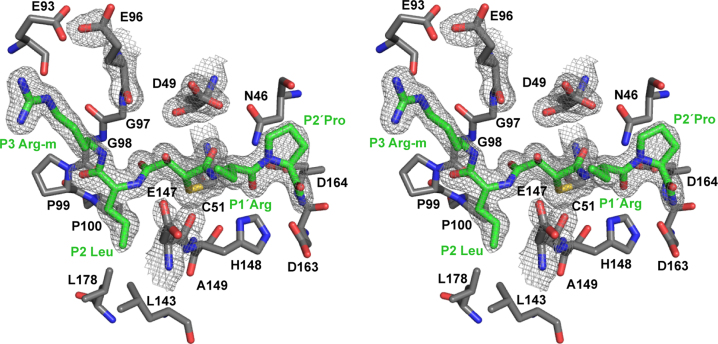
Stereo view of the arrangement of the inhibitor E64-R-P-NH_2_ and the substrate binding site of sLb^pro^. 2F_0_–F_c_ maps contoured at 1 σ are shown as grey mesh for the inhibitor and the sLb^pro^ residues Asp49, Cys51, Glu96 and Glu147. The inhibitor is shown as green sticks. Residues of sLb^pro^ interfacing with the inhibitor are shown as grey sticks. Oxygen, nitrogen and sulphur atoms are coloured red, blue and yellow, respectively. Due to the lack of electron density, no structure is shown for the P1‘ Arg residue of E64-R-P-NH_2_ from the Cδ atom onwards.

**Fig. 4:**
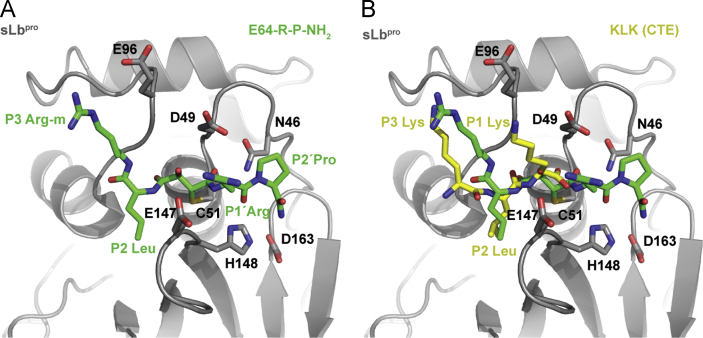
Comparison of the binding of E64-R-P-NH_2_ and P1-P3 of the CTE. (A) The inhibitor (green sticks) is shown in the substrate binding site of sLb^pro^. Side-chains of the inhibitor are labelled. In Fig. 4, Fig. 5, Fig. 6, Fig. 7, the atoms of the P1′ Arg residue from C_δ_ onwards are modelled based on the most favourable conformation. Residues of the active site (Cys51, His148, Asp163) as well as the three acidic residues discussed in the text are shown as sticks. (B) As in A, with the P1-P3 residues (in yellow and labelled) of the CTE superimposed for comparison.

**Fig. 5:**
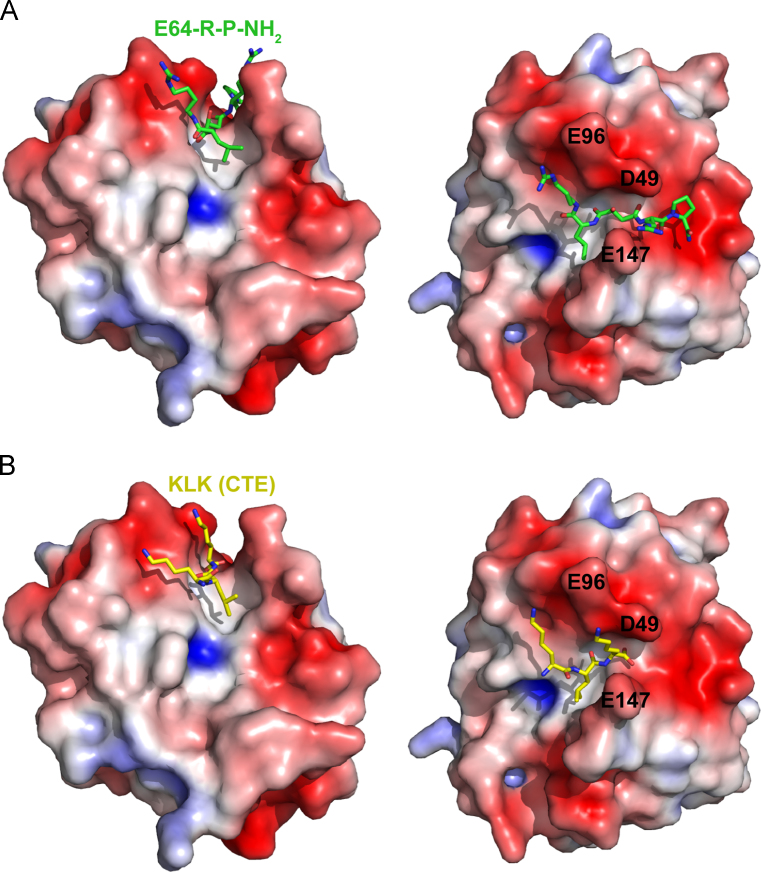
Electrostatic interactions involved in sLb^pro^ interaction with E64-R-P-NH_2_ and the P1-P3 residues of the CTE. The electrostatic potential of sLb^pro^ was calculated using the Adaptive Poisson–Boltzmann Solver package (Baker et al., 2001) within PyMOL (DeLano**,** 2002). The surface is coloured according to the electrostatic potential ranging from −5 (red) to +5 (blue) kT/e. (A) The inhibitor E64-R-P-NH_2_ is shown as green sticks, (B) residues P1-P3 of the CTE as yellow sticks. The representations on the right are rotated 90° on the *x*-axis relative to those on the left.

**Fig. 6:**
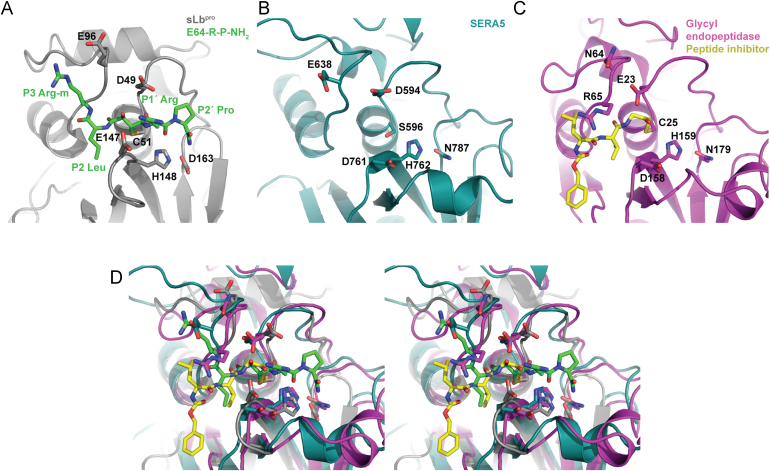
Comparison of arrangement of negatively charged residues in the substrate binding sites of sLb^pro^, glycyl endopeptidase and SERA5. (A) sLb^pro^ bound to E64-R-P-NH_2_ (green sticks). (B) Substrate binding site of SERA5. (C) Glycyl endopeptidase bound to the inhibitor benzyloxycarbonyl-leucine-valine-glycine-methylene (yellow sticks). (D) Stereo view of the superimposition of the three structures in A–C. Residues referred to in the text are shown as sticks.

**Fig. 7:**
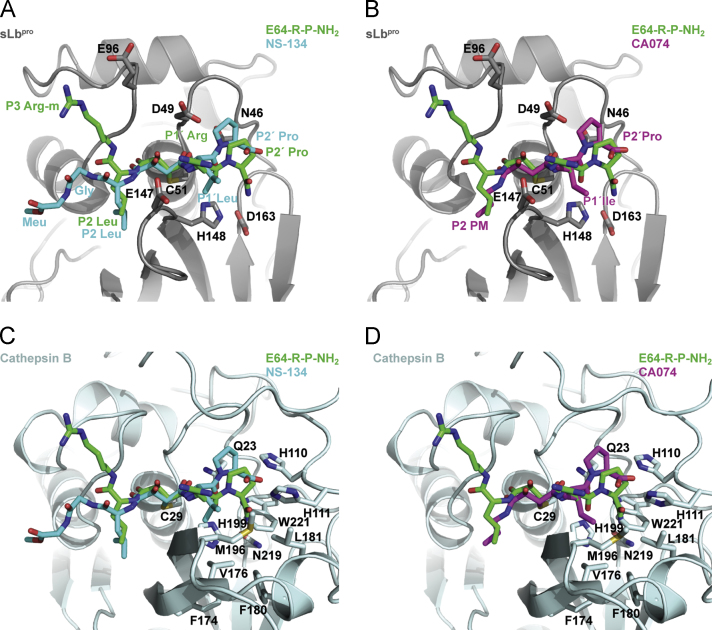
Comparison of the S1′ and S2′ binding sites of FMDV sLb^pro^ and cathepsin B. (A and B) E64-R-P-NH_2_ (green sticks) bound in the substrate binding site of Lb^pro^ with the inhibitors NS-134 (A, blue sticks) and CA074 (B, magenta sticks) superimposed. (C and D) NS-134 (C) and CA074 (D) bound to the substrate binding site of cathepsin B with E64-R-P-NH_2_ superimposed. The colour coding is as in A and B. The structure of Trp221 for which there is no equivalent in Lb^pro^ is shown as sticks as are residues referred to in the text.

The crystal structure of the inhibitor E64-R-P-NH_2_ bound to sLb^pro^ has been determined. The correspondence of the side-chains in the inhibitor to substrate side-chains is illustrated in Fig. 2; a portion of the electron density in the final model of the inhibitor bound to the active site is shown in Fig. 3. Three chains, termed A, B and C were found in the asymmetric unit of the crystal lattice. Electron density was visible for sLb^pro^ residues 29–187 of chain A, 29 to 184 of chain B and 29–185 of chain C. For the inhibitor, electron density was visible for all atoms except for those of the P3 amino-alkyl guanidinium group (referred to as Arg-m in the text and figures) and P1′ Arg. For P3 Arg-m, density was visible up to the *C*_*β*_ atom for chain A, for all atoms of chain B (due to favourable interactions with an Asp residue from a symmetry related molecule) and to atom *N*_*ε*_ for chain C. For the P1′ arginine residues, density up to the C_β_ atom for chain A was visible whereas for chains B and C density was observed to the *C*_*γ*_ atom. The remaining atoms of these side-chains including the guanidinium group were modelled in Fig. 4, Fig. 5, Fig. 6, Fig. 7 after the side-chain trace of *C*_*α*_ to *C*_*γ*_ in the most likely conformation. Density for the covalent bond between the active site cysteine and the inhibitor (atom C1) was very clear in all three chains. Superimposition of the structure of sLb^pro^ bound to E64-R-P-NH_2_ with the unbound Lb^pro^ structure of sLb^pro^ C51A C133S (PDB ID 1QMY, chainB) (Guarné et al., 2000) gave an r.m.s.d. of 0.35 Å over 156*C*_*α*_ atoms superimposed. Given that the best resolution of the inhibitor was found in chain B, all structural analysis is based on this chain.

To determine the binding of E64-R-P-NH_2_ to sLb^pro^, we first compared its arrangement in the substrate binding site of sLb^pro^ to that of the last three residues of the CTE observed in the crystal structure of Lb^pro^ (Guarn**é** et al., 1998). Fig. 4 shows that the positions of the P3 Arg-m side-chain of the inhibitor and the P3 Lys side-chain of the CTE occupy similar positions in the two structures. The *C*_*γ*_ atom of the P1′ Arg residue of the inhibitor lies between the side-chains of Asp49 (distance from *C*_*γ*_ to carboxy group of Asp49 is 4.2 Å) and Glu147 (distance from *C*_*γ*_ to *C*_*β*_ of Glu147 is 4.5 Å). Given the uncertainty in the position of the guanidinium group (as mentioned earlier, the remaining atoms were modelled as no density was observed), a closer localisation is not possible. Nevertheless, the superimposition in Fig. 4B shows that the P1 Lys of the CTE lies almost equidistant between Asp49, Glu96 and Glu147. The disorder of the P1′ Arg in the structure of the inhibitor presented here indicates that the side-chain is flexible; in contrast, in the previously published structure of Lb^pro^ C51A, good density was observed to the P1 Lys residue in the substrate binding site of Lb^pro^ (Guarn**é** et al., 1998). Given that the polypeptide chain is fully extended in both the CTE and E64-R-P-NH_2_ bound structures, this explains how a peptide containing Lys and Arg at P1 and P1′ can be refractory to cleavage (Nogueira Santos et al., 2012). If the Lys at P1 points away from the globular domain, an Arg side-chain at P1′ would have to point towards it. Thus, on oligopeptide substrates at least, the enzyme can only accommodate a basic residue at one of the positions, presumably because it requires a glycine with its greater freedom of rotation at the other. However, the data do not answer the question why a peptide containing Lys and Arg at P1 and P1′ can inhibit Lb^pro^ (Nogueira Santos et al., 2012). This implies that the inhibitor may bind in a mode that has not yet been observed that moves the scissile bond out of the active site. However, additional structural information will be required to elucidate the nature of the binding of this peptide.

Overall, comparison of the binding of the E64-R-P-NH_2_ and the CTE residues (Fig. 5) show that the P1/P1′ binding area is a deep cleft surrounded by the acidic residues Asp49, Glu96 and Glu147. We set out to determine whether other papain-like cysteine proteinases have been identified that have a similar arrangement of three acidic residues in the vicinity of the S1/S1′ binding sites. Berti and Storer (Berti and Storer, 1995) compared the sequences of 48 representative papain-like cysteine proteinases. Only one, SERA5 (Serine repeat antigen 5, termed PfalI in (Berti and Storer, 1995)) from *P. falciparum* showed acidic amino acids at the equivalent positions to those in sLb^pro^; these are Asp594, Glu638 and Asp761 which are equivalent to Asp49, Glu96 and Glu147 of sLb^pro^ ((Hodder et al., 2009); Fig. 6A and B). However, little is known about the biochemistry of this protein; indeed, proteolytic activity has not been shown. Furthermore, the putative active site residue is serine, not cysteine. In addition, the authors suggested that Asp594 (equivalent to Asp49) of SERA5 is too near to the substrate binding site to allow substrate to bind.

A second enzyme, glycyl endopeptidase (ppiv in Berti and Storer, (1995)), also possesses two acidic residues, Glu23 and Asp158, equivalent to Asp49 and Glu147. The third residue (Asn64, equivalent to Glu96 in sLb^pro^) is however not acidic and is followed by Arg65. As can be seen in Fig. 6C, the presence of Glu23 and Arg65 preclude the entry of any substrates with amino acids larger than glycine at P1, thus conferring the specificity referred to in the name glycyl endopeptidase.

It should be noted that only these three papain-like enzymes have an amino acid other than glycine at the position equivalent to Gly23 in papain (equivalent to Asp 49 in sLb^pro^). Superimposition of the three structures (Fig. 6D) shows that Asp49 in sLb^pro^ is further away from the substrate binding site than Glu23 or Asp594 in glycyl endopeptidase (O’Hara et al., 1995) and SERA5 (Hodder et al., 2009). This is due to the presence of only four residues in sLb^pro^ lying between the oxyanion hole defining residue (Asn46) and the active site Cys51. In all other papain-like cysteine proteinases, five residues are present between the oxyanion-hole residue Gln19 and the active site nucleophile Cys25. Interestingly, Glu23 of glycyl endopeptidase is closer to the substrate binding site than Asp594 in SERA5, suggesting that the substrate binding site of SERA5 may be more open than previously thought. In contrast, Glu96 does not superimpose well with Glu638, with the *C*_*α*_ lying 3.7 Å apart (Fig. 6D). Finally, as an important control for the accuracy of the structural superimposition, we note that the *C*_*α*_ of the catalytic histidines (H148, H762 and H159) superimpose well (Fig. 6D).

### Comparisons with inhibitor complexes from cathepsin B

Information on the structural details of the S1′ and S2′ binding sites in papain-like cysteine proteinases is limited, especially for the S2′ site (Turk et al., 1998, Turk et al., 2012). Indeed, structures of compounds with residues bound in the S2′ position are only available for cathepsin B complexed with the inhibitors CA030, CA074 and NS-134 (Fig. 2; (Stern et al., 2004; Turk et al., 1995; Yamamoto et al., 1997)). To compare the binding of the inhibitors CA074 and NS-134 to cathepsin B (CA030 differs only in the length and chemical bond of the N-terminal aliphatic moiety (Turk et al., 1995)) and that of E64-R-P-NH_2_ to sLb^pro^, the structures of cathepsin B complexes with CA074 and NS-134 were superimposed on that of sLb^pro^ using the SSM tool of Coot (Krissinel and Henrick, 2004). The r.m.s.d. values were 2.44 Å for sLb^pro^ superimposed on cathepsin B complexed to CA074 (1QDQ) and 2.31 Å for sLb^pro^ superimposed on cathepsin B complexed to NS-134 (1SP4). Fig. 7A and B show the positions of the inhibitors NS-134 and CA-074 relative to E64-R-P-NH_2_ on sLb^pro^; Fig. 7C and D show the relationships on the structure of cathepsin B.

The P1′ residues of CA074 and NS-134 are Ile and Leu, respectively, in contrast to the Arg found in E64-R-P-NH_2_. Despite the difference in chemical composition, however, the side-chains of these residues superimpose well and occupy the same relative space. The specificity of cathepsin B for these large hydrophobic residues is derived from the presence of a hydrophobic pocket made up of residues Phe174, Val176, Phe180, Leu181, M196 and Trp221 (Figs. 7C and D). In contrast, in sLb^pro^, there is no equivalent loop to those in cathepsin B bearing residues Phe174 to Leu181 and Met196 or Trp221. This region is thus open and, as mentioned before, the presence of Glu147 and Asp49 enable sLb^pro^ to accept well the arginine residue.

At the P2′ position, all three inhibitors have a proline residue. It is clear that the positions of the proline residues from CA074 and NS-134 on the one hand and sLb^pro^ on the other are different. In sLb^pro^, the proline P2′ residue of E64-R-P-NH_2_ lies closer to Asp163, the third member of the catalytic triad. Two factors appear to be responsible for this. The first is the absence of a residue equivalent to Trp221 in cathepsin B (Trp177 in papain) that pushes the proline residue away from Asn219 (equivalent to Asp163 in sLb^pro^). Second, albeit only in chain A, the sLb^pro^ residue Asp164 forms a hydrogen bond (2.7 Å) to the terminal nitrogen on the proline residue whereas in cathepsin B, the terminal carboxyl group of the proline is co-ordinated by His110 and His111 in the occluding loop, a structure unique to cathepsin B that is responsible for its exopeptidase activity. It is clear from the structure that there is no binding pocket for the P2′ residue in sLb^pro^.

### Lb^pro^ and sLb^pro^ differ in cleavage efficiencies on intramolecular substrates

The structural analysis illustrates how sLb^pro^ can bind to an inhibitor bearing Leu, Gly and Arg at the P2, P1 and P1′ sites, respectively, the very residues found at the eIF4GII cleavage site. Nevertheless, a peptide corresponding to the eIF4GI peptide (SFANLG⁎RTTL) was a poor substrate for both Lb^pro^ and sLb^pro^ ((Santos et al., 2009); unpublished data). However, the eIF4GI cleavage site when introduced into the background of the polyprotein substrate was efficiently cleaved by Lb^pro^ but was still refractory to cleavage by sLb^pro^ (Cencic et al., 2007). Examination of the state of the endogenous eIF4GI in RRLs used for the experiments showed that it was cleaved by both Lb^pro^ and sLb^pro^ (Cencic et al., 2007).

To understand these observations and illuminate differences in cleavage efficiencies between Lb^pro^ and sLb^pro^, we decided to investigate further the cleavage of intermolecular polyprotein substrates using the system described by Cencic et al. (2007). Here, a fragment of the FMDV polyprotein encoding an inactive form of Lb^pro^, VP4 and part of VP2 (termed Lb^pro^ C51A VP4/VP2) is labelled with ^35^S methionine by translation in RRLs (Cencic et al., 2007). Subsequently, cold methionine is added and an mRNA encoding an active, mature Lb^pro^ or sLb^pro^ is added. The enzyme was synthesised from an RNA molecule rather than adding purified recombinant proteinase for two reasons. First, preparations of purified active Lb^pro^ always contain some sLb^pro^ that arises from self-processing, even when all purification steps are done at 4 °C (Kirchweger et al., 1994). Second, the translation of the RNA followed by Lb^pro^ cleavage of the eIF4G isoforms in the RRLs resembles more closely the in vivo situation during an FMDV infection. The labelled substrate and products are separated by SDS-PAGE and detected by fluorography. Although it is very difficult to vary either the enzyme or substrate concentrations in this assay, it still provides qualitative information on differences in the rates of reaction between different forms of L^pro^.

**Fig. 8:**
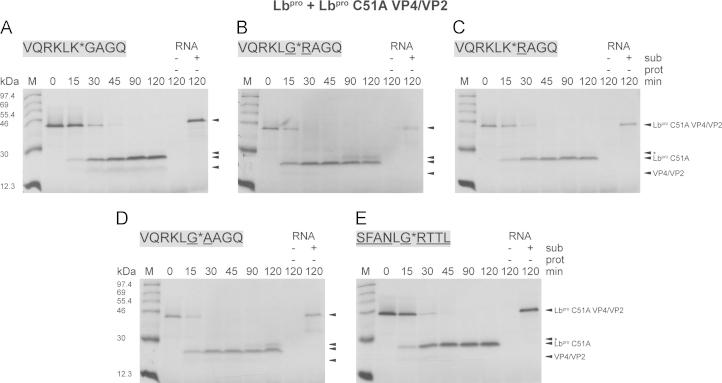
Effects of P1 or P1′ site mutations on the intermolecular cleavage efficiency of Lb^pro^. Intermolecular processing of the precursor Lb^pro^ C51A VP4/VP2 (A) and variants thereof (B–E) by Lb^pro^. The cleavage sequence present in the background of the polyprotein is shown in grey boxes. Differences from the wild-type sequence of the precursor are underlined. RRLs were programmed with RNA (14 ng/µl) coding for the polyprotein Lb^pro^ C51A VP4/VP2. Translation was performed for 20 min at 30 °C in the presence of [^35^S]-Met and terminated by the addition of unlabelled Met for 10 min. RNA (14 ng/µl) coding for Lb^pro^ was added and translation was continued at 30 °C. The reaction was terminated by placing the samples on ice and the addition of Laemmli sample buffer containing excess unlabelled Met and Cys. 10 µl aliquots were analysed by 17.5% SDS-PAGE gels, followed by fluorography. Uncleaved precursor Lb^pro^ C51A VP4/VP2 and cleavage products Lb^pro^ C51A and VP4/VP2 are indicated. The asterisk (*) indicates an aberrant cleavage product. Negative controls devoid of any RNA (-sub, -prot) or comprising only RNA encoding the precursor (+sub, -prot) are shown on the right of each gel. Protein standards are shown on the left. Each cleavage reaction was performed twice; a representative autoradiogram for each is shown.

A typical experiment is shown in Fig. 8A, with Lb^pro^ cleaving the wild-type sequence between 15 and 30 min. The Lb^pro^ moiety has four methionine residues compared to only two in the VP4/VP2 part, providing a partial explanation for the lower intensity of the latter band (Glaser et al., 2001). In addition, we have evidence that the VP4/VP2 part is degraded in the RRLs (data not shown), with degradation being enhanced when a residue other than the wild-type glycine is present at the N-terminus of VP4 (see Fig. 8B–E). We first investigated the effect of substituting residues at the P1 and P1′ positions with residues (underlined in Fig. 8B–D), several of which had been shown to be detrimental to peptide cleavage (Guarné et al., 2000, Nogueira Santos et al., 2012, Santos et al., 2009). However, none of the modifications affected the cleavage efficiency of Lb^pro^ (Table 2). These results show that there are clear differences between the activity of Lb^pro^ on peptides and polyprotein substrates, indicating that the conformation of the substrate may be different in the background of the polyprotein. In addition, as previously observed, replacement of the P5-P4′ residues of the polyprotein cleavage sequence with those of the eIF4GI site (Fig. 8E) also did not affect the efficiency of Lb^pro^ cleavage.

**Table 2:** Summary of the mutational analysis of the intermolecular cleavage efficiency of Lb^pro^ and sLb^pro^. Data are taken from Fig. 8, Fig. 9 and Glaser et al., 2001 for cleavage of eIF4GI by Lb^pro^. The experiments were performed twice.

	**50% cleavage (min)**					
	**pCITE Lb**^**pro**^**C51A VP4/VP2**					**endogenous eIF4GI**
	**VQRKLK^⁎^GAGQ**	**VQRKLG^⁎^RAGQ**	**VQRKLK^⁎^RAGQ**	**VQRKLG^⁎^AAGQ**	**SFANLG^⁎^RTTL (eIF4GI)**	**SFANLG^⁎^RTTL**
**Lb**^**pro**^	**15–30**	**0**–**15**	**15**–**30**	**0**–**15**	**15**–**30**	**0**–**15**
**sLb**^**pro**^	**30**	**30**	**45**–**90**	**90**	**No cleavage**	**0**–**15**

**Fig. 9:**
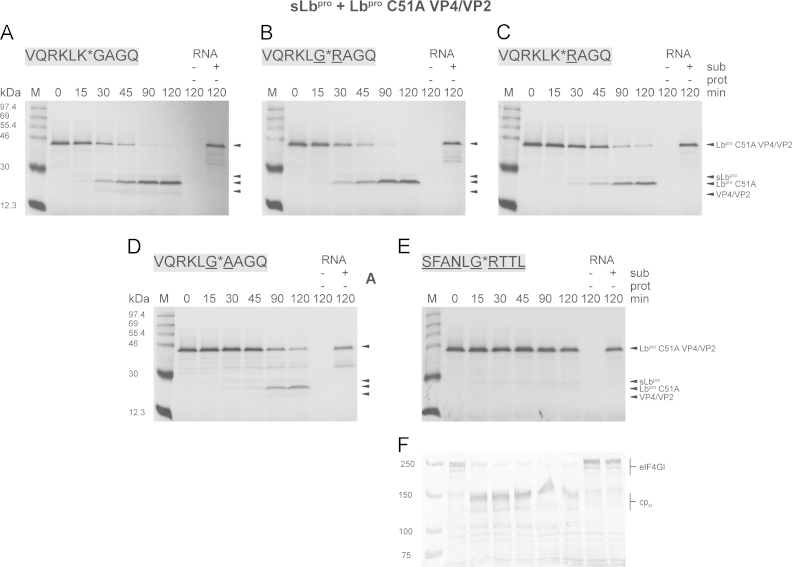
Effects of P1 or P1′ site mutations on the intermolecular cleavage efficiency of sLb^pro^. The intermolecular processing of the precursor Lb^pro^ C51A VP4/VP2 (A) and variants thereof (B–E) by sLb^pro^. The cleavage sequence present in the background of the polyprotein is shown in grey boxes. Differences from the wild-type sequence of the precursor are underlined. The translation and analysis was done as shown in Fig. 8. (F) The intermolecular cleavage of endogenous eIF4GI present in the RRLs from panel E. 10 µl aliquots were analysed on a 6% Dasso & Jackson SDS-PAGE (Dasso and Jackson, 1989), followed by immunoblotting with an antiserum detecting the N-terminal part of eIF4GI. Uncleaved eIF4GI and cleavage products (cp_N_) are indicated. Negative controls devoid of any RNA (−sub, −prot) or comprising only RNA encoding the precursor (+sub, −prot) are shown. Protein standards are shown on the left. Each cleavage reaction was performed twice; a representative autoradiogram for each is shown.

We next investigated the ability of sLb^pro^ to cleave the same five substrates. In all cases, sLb^pro^ cleavage was delayed compared to the Lb^pro^ cleavage. 50% cleavage of the wild-type substrate and a substrate bearing P1 Gly and P1′ Arg occurred at 30 min (Fig. 9A and B) compared to 15–30 min and 0–15 min, respectively, with Lb^pro^ (Table 1). 50% cleavage of the substrate bearing two basic amino acids at the scissile bond was observed between 45–90 min whereas the substrate lacking basic amino acids at the cleavage site only showed 50% cleavage after 90 min. Finally, as was shown previously by Cencic et al. (2007), the substrate with the eIF4GI cleavage site was not cleaved at all, even after 120 min of incubation. Thus, the cleavage efficiencies of sLb^pro^ on modified polyprotein substrates are similar to those on analogously modified oligopeptides, in contrast to those of Lb^pro^. As a control, we examined the state of the endogenous eIF4GI present in RRLs by performing a Western blot of the cleavage reaction using an anti-eIF4GI antiserum (Fig. 9F). Endogenous eIF4GI was cleaved within 15 min after the start of incubation, an efficiency comparable to that previously observed with Lb^pro^ and sLb^pro^ (Glaser et al., 2001). This cleavage efficiency on endogenous eIF4GI was also observed in all other reactions in Fig. 9 (data not shown).

**Fig. 10:**
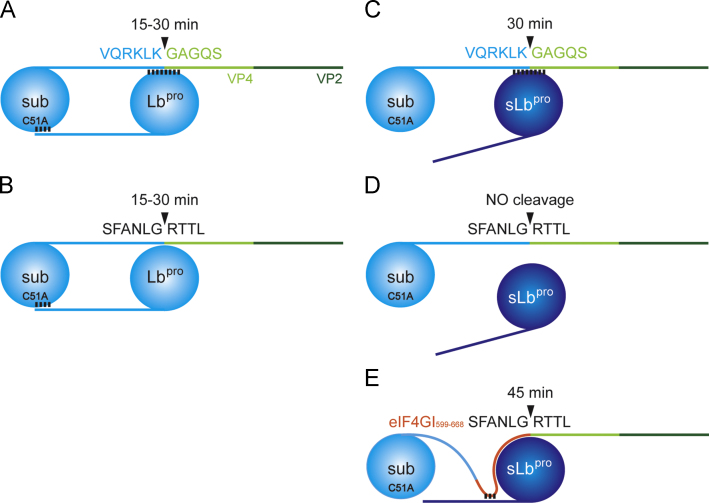
Model of the intermolecular cleavage of polyprotein substrates by Lb^pro^ and sLb^pro^. (A and C) Cleavage of wild-type Lb^pro^ C51A VP4/VP2 by Lb^pro^ and sLb^pro^, respectively. (B and D) Cleavage of Lb^pro^ C51A VP4/VP2 containing the eIF4GI cleavage sequence by Lb^pro^ and sLb^pro^, respectively. E. Cleavage of Lb^pro^ C51A eIF4GI_599-668_ VP4/VP2 SFANLG*RTTL by sLb^pro^. Lb^pro^ is in light blue, sLb^pro^ in dark blue, VP4 in light green, VP2 in dark green and the eIF4GI_599-668_ fragment in red.

How can we explain the differences observed above between Lb^pro^ and sLb^pro^ in their behaviour towards oligopeptide and polyprotein substrates (Table 2)? Why do the introduced mutations only affect sLb^pro^ cleavage and why is sLb^pro^ not capable of recognising the eIF4GI cleavage site in the background of the polyprotein? The difference in the cleavage efficiencies between Lb^pro^ and sLb^pro^ on polyprotein protein substrates can be explained by the ability of Lb^pro^ to bind to the cleavage site on the substrate with its canonical substrate binding site and through its own CTE to the “substrate binding site” of the substrate as shown in Fig. 10A and B. In contrast, sLb^pro^ can only make one of these interactions, as it lacks an intact CTE (Fig. 10C and D).

**Fig. 11:**
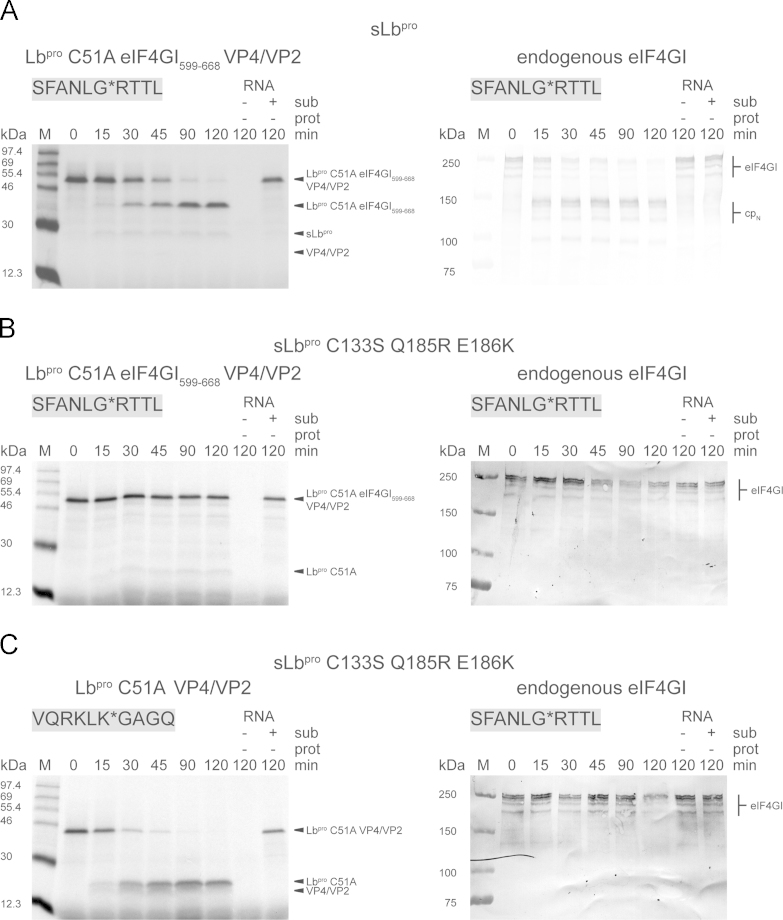
eIF4GI_599-668_ is essential for the cleavage of the sub-optimal eIF4GI cleavage site SFANLG*RTTL by sLb^pro^. A-C, left panels. Intermolecular processing of Lb^pro^ C51A eIF4GI_599-668_ VP4/VP2 by sLb^pro^ and sLb^pro^ C133S Q185R E186K and Lb^pro^ C51A VP4/VP2 by sLb^pro^ C133S Q185R E186K. The cleavage sequence present in the background of the polyprotein is shown in grey boxes. Translation reactions and analyses of products were as in Fig. 8. Uncleaved precursors Lb^pro^ C51A eIF4GI_599-668_ VP4/VP2 and Lb^pro^ C51A VP4/VP2 as well as cleavage products Lb^pro^ C51A eIF4GI_599-668_, Lb^pro^ C51A and VP4/VP2 are indicated. A–C, right panels. The intermolecular cleavage of endogenous eIF4GI present in the RRL. Analysis of the state of eIF4GI was as shown in Fig. 9. Uncleaved eIF4GI and cleavage products (cp_N_) are indicated. Negative controls lacking added RNA (−sub, −prot) or comprising only of RNA encoding the precursor (+sub, −prot) are shown. Protein standards are shown on the left.

sLb^pro^ can efficiently cleave the eIF4GI site on the native protein present in the RRL because this involves residues Cys133 and Asp184-Leu188 of the CTE but not the last six residues of the CTE (Foeger et al., 2005). Accordingly, we introduced 80 amino acids from eIF4GI containing the L^pro^ binding and cleavage sites into the Lb^pro^ C51A VP4/VP2 substrate (Fig. 10E). This modified substrate (termed Lb^pro^ C51A eIF4GI_599-668_ VP4/VP2 SFANLG⁎RTTL) was cleaved by sLb^pro^ between 30 and 90 min, indicating that the availability of the two binding sites had allowed cleavage to take place (Fig. 11A).

As a control, we examined the ability of the variant sLb^pro^ C133S Q185R E186K that had previously been shown to be unable to cleave endogenous eIF4GI because the variant cannot recognise its binding site on this factor (Foeger et al., 2005). This variant could also not cleave the Lb^pro^ C51A eIF4GI_599-668_ VP4/VP2, but maintains the ability to cleave the wild-type substrate Lb^pro^ C51A VP4/VP2 (Figs. 11B and C, left panels). As previously reported, sLb^pro^ C133S Q185R E186K was however not able to cleave endogenous eIF4GI, even after 120 min of incubation (Figs. 11B and C, right panels).

## Concluding remarks

The structural data presented here reveal that sLb^pro^ uses three acidic residues to bind to basic residues at the P1 or P1′ positions of a substrate and that this represents a unique arrangement that is not found in cellular papain-like proteinases. Differences in the cleavage efficiency of Lb^pro^ and sLb^pro^ were observed on modified polyprotein substrates. The presence of sLb^pro^ in infected cells (Sangar et al., 1988) suggests that differences in the properties of Lb^pro^ and sLb^pro^ will be relevant to the success of viral replication. Hence, the removal of six C-terminal residues 40 Å from the active site may represent a unique mechanism to modify the properties of a proteolytic enzyme during viral replication.

## References

[bib1] Adams P.D., Afonine P.V., Bunkoczi G., Chen V.B., Davis I.W., Echols N., Headd J.J., Hung L.W., Kapral G.J., Grosse-Kunstleve R.W., McCoy A.J., Moriarty N.W., Oeffner R., Read R.J., Richardson D.C., Richardson J.S., Terwilliger T.C., Zwart P.H. (2010).

[bib2] Baker N.A., Sept D., Joseph S., Holst M.J., McCammon J.A. (2001). Electrostatics of nanosystems: application to microtubules and the ribosome. Proc. Natl. Acad. Sci. USA.

[bib3] Berti P.J., Storer A.C. (1995). Alignment/phylogeny of the papain superfamily of cysteine proteases. J. Mol. Biol..

[bib4] Cao X., Bergmann I.E., Fullkrug R., Beck E. (1995). Functional analysis of the two alternative translation initiation sites of foot-and-mouth disease virus. J. Virol..

[bib5] Cencic R., Mayer C., Juliano M.A., Juliano L., Konrat R., Kontaxis G., Skern T. (2007). Investigating the substrate specificity and oligomerisation of the leader protease of foot and mouth disease virus using NMR. J. Mol. Biol..

[bib6] Dasso M.C., Jackson R.J. (1989). Efficient initiation of mammalian mRNA translation at a CUG codon. Nucleic Acids Res..

[bib7] Davis I.W., Leaver-Fay A., Chen V.B., Block J.N., Kapral G.J., Wang X., Murray L.W., Arendall W.B., Snoeyink J., Richardson J.S., Richardson D.C. (2007). MolProbity: all-atom contacts and structure validation for proteins and nucleic acids. Nucleic Acids Res..

[bib8] de Los Santos T., Diaz-San Segundo F., Grubman M.J. (2007). Degradation of nuclear factor kappa B during foot-and-mouth disease virus infection. J. Virol..

[bib9] de los Santos T., Segundo F.D., Zhu J., Koster M., Dias C.C., Grubman M.J. (2009). A conserved domain in the leader proteinase of foot-and-mouth disease virus is required for proper subcellular localization and function. J. Virol..

[bib10] DeLano W.L. (2002). The PyMOL Molecular Graphics System.

[bib11] Devaney M.A., Vakharia V.N., Lloyd R.E., Ehrenfeld E., Grubman M.J. (1988). Leader protein of foot-and-mouth disease virus is required for cleavage of the p220 component of the cap-binding protein complex. J. Virol..

[bib12] Emsley P., Cowtan K. (2004). Coot: model-building tools for molecular graphics. Acta Crystallogr. D Biol. Crystallogr..

[bib13] Etchison D., Milburn S.C., Edery I., Sonenberg N., Hershey J.W.B. (1982). Inhibition of HeLa cell protein synthesis following poliovirus infection correlates with the proteolysis of a 220,000-dalton polypeptide associated with eucaryotic initiation factor 3 and a cap binding protein complex. J. Biol. Chem..

[bib14] Foeger N., Kuehnel E., Cencic R., Skern T. (2005). The binding of foot-and-mouth disease virus leader proteinase to eIF4GI involves conserved ionic interactions. FEBS J..

[bib15] Gingras A.C., Raught B., Sonenberg N. (1999). eIF4 initiation factors: effectors of mRNA recruitment to ribosomes and regulators of translation. Annu. Rev. Biochem..

[bib16] Glaser W., Cencic R., Skern T. (2001). Foot-and-mouth disease leader proteinase: involvement of C-terminal residues in self-processing and cleavage of eIF4GI. J. Biol. Chem..

[bib17] Gradi A., Foeger N., Strong R., Svitkin Y.V., Sonenberg N., Skern T., Belsham G. (2004). Cleavage of eukaryotic translation initiation factor 4GII within foot-and-mouth disease virus-infected cells: identification of the L-protease cleavage site in vitro. J. Virol..

[bib19] Guarné A., Hampoelz B., Glaser W., Carpena X., Tormo J., Fita I., Skern T. (2000). Structural and biochemical features distinguish the foot-and-mouth disease virus leader proteinase from other papain-like enzymes. J. Mol. Biol..

[bib20] Guarné A., Tormo J., Kirchweger K., Pfistermueller D., Fita I., Skern T. (1998). Structure of the foot-and-mouth disease virus leader protease: a papain-like fold adapted for self-processing and eIF4G recognition. EMBO J..

[bib21] Hodder A.N., Malby R.L., Clarke O.B., Fairlie W.D., Colman P.M., Crabb B.S., Smith B.J. (2009). Structural insights into the protease-like antigen *Plasmodium falciparum* SERA5 and its noncanonical active-site serine. J. Mol. Biol..

[bib22] Kabsch W. (2010). Xds. Acta Crystallogr. D Biol. Crystallogr..

[bib23] Kirchweger R., Ziegler E., Lamphear B.J., Waters D., Liebig H.D., Sommergruber W., Sobrino F., Hohenadl C., Blaas D., Rhoads R.E., Skern T. (1994). Foot-and-mouth disease virus leader proteinase: purification of the Lb form and determination of its cleavage site on eIF-4 gamma. J. Virol..

[bib24] Kräusslich H.G., Nicklin M.J., Toyoda H., Etchison D., Wimmer E. (1987). Poliovirus proteinase 2A induces cleavage of eucaryotic initiation factor 4F polypeptide p220. J. Virol..

[bib25] Krissinel E., Henrick K. (2004). Secondary-structure matching (SSM), a new tool for fast protein structure alignment in three dimensions. Acta Crystallogr. D Biol. Crystallogr..

[bib26] Lamphear B.J., Kirchweger R., Skern T., Rhoads R.E. (1995). Mapping of functional domains in eukaryotic protein synthesis initiation factor 4 G (eIF4G) with picornaviral proteases - implications for cap-dependent and cap- independent translational initiation. J. Biol. Chem..

[bib27] Leibowitz R., Penman S. (1971). Regulation of protein synthesis in HeLa cells. 3. Inhibition during poliovirus infection. J. Virol..

[bib28] Luft J.R., DeTitta G.T. (1999). A method to produce microseed stock for use in the crystallisation of biological macromolecules. Acta Crystallogr. D Biol. Crystallogr..

[bib29] Martinez-Salas E., Ryan M.D., Ehrenfeld E., Domingo E., Roos R.P. (2010). The Picornaviruses.

[bib30] Mayer C., Neubauer D., Nchinda A.T., Cencic R., Trompf K., Skern T. (2008). Residue L143 of the foot-and-mouth disease virus leader proteinase is a determinant of cleavage specificity. J. Virol..

[bib31] Murshudov G.N., Vagin A.A., Dodson E.J. (1997). Refinement of macromolecular structures by the maximum-likelihood method. Acta Crystallogr. D Biol. Crystallogr..

[bib32] Neubauer D., Aumayr M., Gosler I., Skern T. (2013). Specificity of human rhinovirus 2A(pro) is determined by combined spatial properties of four cleavage site residues. J. Gen. Virol..

[bib33] Nogueira Santos J.A., Assis D.M., Gouvea I.E., Judice W.A., Izidoro M.A., Juliano M.A., Skern T., Juliano L. (2012). Foot and mouth disease leader protease (Lbpro): Investigation of prime side specificity allows the synthesis of a potent inhibitor. Biochimie.

[bib34] O׳Hara B.P., Hemmings A.M., Buttle D.J., Pearl L.H. (1995). Crystal structure of glycyl endopeptidase from Carica papaya: a cysteine endopeptidase of unusual substrate specificity. Biochemistry.

[bib35] Petersen J.F., Cherney M.M., Liebig H.D., Skern T., Kuechler E., James M.N. (1999). The structure of the 2A proteinase from a common cold virus: a proteinase responsible for the shut-off of host-cell protein synthesis. EMBO J..

[bib36] Sangar D.V., Clark R.P., Carroll A.R., Rowlands D.J., Clarke B.E. (1988). Modification of the leader protein (Lb) of foot-and-mouth disease virus. J. Gen. Virol..

[bib37] Sangar D.V., Newton S.E., Rowlands D.J., Clarke B.E. (1987). All foot and mouth disease virus serotypes initiate protein synthesis at two separate AUGs. Nucleic Acids Res..

[bib38] Santos J.A., Gouvea I.E., Judice W.A., Izidoro M.A., Alves F.M., Melo R.L., Juliano M.A., Skern T., Juliano L. (2009). Hydrolytic properties and substrate specificity of the foot-and-mouth disease leader protease. Biochemistry.

[bib39] Schechter I., Berger A. (1967). On the size of the active site in proteases. I. Papain. Biochem. Biophys. Res. Commun..

[bib40] Skern T., Steinberger J. (2014). The leader proteinase of foot-and-mouth disease virus: structure-function relationships in a proteolytic virulence factor. Biol. Chem..

[bib41] Steinberger J., Kontaxis G., Rancan C., Skern T. (2013). Comparison of self-processing of foot-and-mouth disease virus leader proteinase and porcine reproductive and respiratory syndrome virus leader proteinase nsp1alpha. Virology.

[bib42] Stern I., Schaschke N., Moroder L., Turk D. (2004). Crystal structure of NS-134 in complex with bovine cathepsin B: a two-headed epoxysuccinyl inhibitor extends along the entire active-site cleft. Biochem. J..

[bib43] Strebel K., Beck E. (1986). A second protease of foot-and mouth disease virus. J. Virol..

[bib44] Turk D., Guncar G., Podobnik M., Turk B. (1998). Revised definition of substrate binding sites of papain-like cysteine proteases. Biol. Chem..

[bib45] Turk D., Podobnik M., Popovic T., Katunuma N., Bode W., Huber R., Turk V. (1995). Crystal structure of cathepsin B inhibited with CA030 at 2.0-A resolution: a basis for the design of specific epoxysuccinyl inhibitors. Biochemistry.

[bib46] Turk V., Stoka V., Vasiljeva O., Renko M., Sun T., Turk B., Turk D. (2012). Cysteine cathepsins: from structure, function and regulation to new frontiers. Biochim. Biophys. Acta.

[bib47] Winn M.D., Ballard C.C., Cowtan K.D., Dodson E.J., Emsley P., Evans P.R., Keegan R.M., Krissinel E.B., Leslie A.G., McCoy A., McNicholas S.J., Murshudov G.N., Pannu N.S., Potterton E.A., Powell H.R., Read R.J., Vagin A., Wilson K.S. (2011). Overview of the CCP4 suite and current developments. Acta Crystallogr. D Biol. Crystallogr..

[bib48] Yamamoto A., Hara T., Tomoo K., Ishida T., Fujii T., Hata Y., Murata M., Kitamura K. (1997). Binding mode of CA074, a specific irreversible inhibitor, to bovine cathepsin B as determined by X-ray crystal analysis of the complex. J. Biochem..

[bib49] Zhou Q., Krebs J.F., Snipas S.J., Price A., Alnemri E.S., Tomaselli K.J., Salvesen G.S. (1998). Interaction of the baculovirus anti-apoptotic protein p35 with caspases. Specificity, kinetics, and characterization of the caspase/p35 complex. Biochemistry.

